# Mechanistic insights into cadmium-induced hepatotoxicity mediated by dysregulation of microRNA expression

**DOI:** 10.3389/fcell.2026.1787688

**Published:** 2026-02-19

**Authors:** Runcen Xu, Yiming Liu, Rui Gao, Zhifeng Zhang, Ye Feng, Yuan Yuan, Zhi Chen, Le Hu

**Affiliations:** 1 Faculty of Medicine, Yangzhou University, Yangzhou, China; 2 College of Social Development, Yangzhou University, Yangzhou, China; 3 College of Animal Science and Technology, Yangzhou University, Yangzhou, China; 4 School of Nursing, Faculty of Medicine, Yangzhou University, Yangzhou, China; 5 Northern Jiangsu People’s Hospital Affiliated to Yangzhou University, Yangzhou, China; 6 The Yangzhou School of Clinical Medicine of Dalian Medical University, Yangzhou, China

**Keywords:** cadmium (Cd), inflammation, liver, miRNA, signal transduction

## Abstract

Cadmium (Cd), a toxic heavy metal pollutant, poses a serious threat to environmental and health and exhibits pronounced hepatotoxicity. However, the underlying mechanisms—particularly those involving microRNA (miRNA) regulation—remain incompletely understood. In this study, an acute liver injury model was established in mice via oral gavage administration of cadmium chloride (18 μg/L, 0.8 mL/day). A comprehensive methodological approach was employed, including serum biochemical assays, histopathological examination, transmission electron microscopy, quantitative Polymerase Chain Reaction acronym (PCR), and high-throughput miRNA sequencing combined with bioinformatic analyses, to systematically investigate the mechanisms of Cd-induced hepatotoxicity. The results demonstrated that Cd exposure led to marked hepatic injury, reflected by altered liver function indices, hepatocellular ultrastructural disruption, apoptosis, and inflammatory responses. However, these downstream pathological changes do not explain how the coordinated inflammatory and apoptotic responses are initiated at the molecular level, highlighting the need to identify upstream regulatory mechanisms. To explore upstream regulatory mechanisms, miRNA transcriptomic analysis indicated that the target genes of differentially expressed miRNAs are enriched in inflammation- and apoptosis-related pathways, including MAPK and TNF signaling. These results suggest a potential role for miRNA-mediated regulation in cadmium-induced liver injury in mice and provide a basis for further investigation of molecular responses to heavy metal exposure.

## 1 Introduction

Heavy metal contamination has become an increasingly severe environmental problem, attracting global concern due to its adverse effects on ecosystems and human health (Briffa et al., 2020). Heavy metals, including cadmium (Cd), mercury (Hg), manganese (Mn), lead (Pb), and selenium (Se), are widely distributed in the environment (Kim et al., 2019). Although some of these metals are essential for biological processes, excessive exposure can lead to significant toxicity. Cadmium, in particular, is extensively used in industrial applications and released as a byproduct of mining, metal smelting, and fossil fuel combustion, making it a major contributor to environmental pollution (Briffa et al., 2020; Han et al., 2022). Cd contamination has been documented in air, water, and soil, posing health risks through inhalation, dermal absorption, and especially ingestion (Munir et al., 2022; Bhardwaj et al., 2023; Phan and Nguyen, 2018; Nguyen and Huynh, 2023). Due to its persistence and strong bioaccumulation, Cd is not easily eliminated from the body and primarily accumulates in organs such as the liver, which is a major site of cadmium deposition and a target for hepatotoxicity (Zhu et al., 2021; European Food Safety Autho rity, 2017). Epidemiological studies have linked even low-level Cd exposure to cardiovascular diseases (Xu et al., 2021), renal dysfunction (Hagedoorn et al., 2020), and osteoporosis (Ma et al., 2021).

Experimental studies indicate that Cd exerts toxicity through multiple mechanisms. Its high affinity for sulfhydryl (-SH) groups allows it to bind proteins, enzymes, and nucleic acids, disrupting their structure and function (European Food Safety Autho rity, 2017; Nordberg et al., 2018). Cd exposure also induces oxidative stress by increasing reactive oxygen species (ROS) production or impairing antioxidant defenses, including catalase, MnSOD, and CuZnSOD (Matović et al., 2015; Wang et al., 2009). Furthermore, Cd disrupts mitochondrial homeostasis, affecting the balance between fission and fusion, thereby promoting apoptosis and necrosis (Xu et al., 2013). As demonstrated by the above findings, cadmium exposure primarily impairs hepatocellular physiological functions by inducing oxidative stress, inflammatory responses, and dysregulation of gene expression, thereby promoting the onset and progression of hepatotoxicity. Despite these insights into downstream effects such as oxidative stress, inflammation, and cell death, the upstream molecular regulatory networks governing Cd-induced hepatotoxicity remain poorly understood.

MicroRNAs (miRNAs) are endogenous, single-stranded, non-coding RNAs of approximately 22 nucleotides that regulate gene expression post-transcriptionally by binding target mRNAs, leading to mRNA degradation or translational repression (Bartel, 2018; Iwasaki et al., 2010; Ha and Kim, 2014; Zeng et al., 2025). Recent evidence suggests that miRNAs are involved in Cd-induced liver injury. For instance, exposure of HepG2 cells to 10 μM Cd for 24 h led to downregulation of 12 miRNAs, including let-7a, let-7b, let-7e, let-7g, and miR-455-3p (Fabbri et al., 2012). These miRNAs commonly function in the body to degrade target mRNAs, inhibit their translation, suppress cell proliferation and inflammation, and promote apoptosis. Target gene analysis revealed enrichment in focal adhesion and MAPK signaling pathways, indicating that miRNAs may modulate Cd-induced hepatotoxicity through specific biological processes (Fabbri et al., 2012). However, comprehensive studies employing high-throughput miRNA profiling to systematically identify Cd-responsive miRNAs and their regulatory networks *in vivo* are still lacking.

Despite growing evidence that Cd disrupts hepatic function, a comprehensive understanding of the upstream regulatory mechanisms, particularly the role of miRNAs, remains lacking. This study aimed to systematically elucidate the mechanisms underlying cadmium-induced acute liver injury by establishing an acute liver injury model and performing high-throughput transcriptomic analysis to profile the global expression of microRNAs in the liver. We further sought to identify key Cd-responsive miRNAs and their target gene networks that may mediate hepatotoxicity. We hypothesize that acute Cd exposure induces specific changes in hepatic miRNA expression, which in turn regulate critical molecular pathways driving liver injury.

## 2 Materials and methods

### Ethics statement

The experimental procedures received authorization from both the Administration of Affairs Concerning Experimental Animals of the Ministry of Science and Technology of China (2025) and the Institutional Animal Care and Use Committee of the College of Animal Science and Technology at Yangzhou University. When adult animals needed to be euthanized, humane methods were employed under standard conditions to minimize any potential discomfort or distress.

### 2.1 Experimental animals and sample collection

C57 mice (6–7 weeks old) were housed under controlled environmental conditions at a temperature of 21 °C ± 2 °C and relative humidity of 60%–70%, with a 12 h light/dark cycle. Animals had free access to standard chow and water throughout the experiment. Mice were randomly assigned to receive either vehicle (sterile saline) or cadmium (Cd) in the vehicle solution via oral gavage. To determine the optimal Cd exposure concentration for inducing hepatic injury, mice were administered CdCl_2_ at concentrations of 1.5, 3, 10, 18, 50, 90, 250, 450, and 1,200 μg/L (0.8 mL/day) for 5 days. Each group consisted of three mice, with an additional control group (n = 3) receiving sterile saline. Among these groups, exposure to 18 μg/L CdCl_2_ resulted in the most pronounced hepatic injury. Based on this finding, subsequent experiments were designed to evaluate the time-dependent effects of Cd exposure. Mice were divided into four groups: pre-exposure (day 0), 3-day exposure (18 μg/L, 0.8 mL/day), 5-day exposure (18 μg/L, 0.8 mL/day), and 7-day exposure (18 μg/L, 0.8 mL/day), with three mice per group. To further assess tissue-specific responses to Cd exposure, mice were treated with high, medium, and low concentrations of CdCl_2_ (0.8 mL/day) for 5 days, with three mice per group. Following exposure, mice were deeply anesthetized with sodium pentobarbital (100 mg/kg body weight, intraperitoneally; Butler Co., Columbus, OH). Blood was collected via abdominal aorta transection, and organs and tissues were immediately harvested for further analysis.

### 2.2 Biochemical and hematological assays

The serum biochemical parameters of mice were measured by using an animal biochemical analyzer (SMT-120VP, Seamaty, CHINA) and animal biochemical reagent plate (Seamaty, CHINA). The concentrations of Aspartate aminotransferase (AST), Blood urea nitrogen (BUN), Glucose (GLU), Total cholesterol (CHOL), Triglycerides (TG), High-density lipoprotein cholesterol (HDL-C), White Blood Cell Count (WBC), Hemoglobin (HGB), Platelet Count (PLT), Lymphocyte Count (Lymph#), Red Cell Distribution Width (RDW), Mean Platelet Volume (MPV), in serum were measured in the present study.

### 2.3 Hematoxylin and eosin (H&E) staining of cells

Treated hepatocytes were first dewaxed in xylene for 10 min, after which the reagent was removed using 100% ethanol. The cells were then rehydrated through a graded ethanol series (95%, 85%, and 75%) for 5 min each. Hematoxylin was applied for a 3-min stain, followed by a 2-min rinse in distilled water. The sections were subsequently subjected to brief differentiation in 1% hydrochloric acid alcohol for 2 s and then washed in distilled water for 15 min. A drop of neutral gum was used to mount the slides. Finally, the prepared sections were examined and imaged under a microscope.

### 2.4 Immunohistochemistry

The immunohistochemistry procedure followed the approach described by Soundararajan et al. (2022). Slides were incubated at 60 °C for 30–60 min, then dewaxed in xylene and rehydrated through a graded ethanol series. Antigen retrieval was performed in citrate buffer (pH 6.0) using microwave heating, followed by cooling and PBS washes. Endogenous peroxidase activity was blocked with 3% H_2_O_2_, and sections were blocked with 5% goat serum. Sections were incubated overnight at 4 °C with primary antibody (PBS for control), followed by secondary antibody incubation at 37 °C. Signal was developed with DAB, and sections were counterstained with hematoxylin. Finally, sections were dehydrated, cleared in xylene, mounted with neutral resin, and observed under a microscope.

### 2.5 TUNEL experiment

Liver sections were dewaxed in xylene (2 × 10 min) and rehydrated through graded ethanol solutions (100%, 95%, 85%, and 75%) before rinsing in distilled water. Antigen retrieval and permeabilization were performed using proteinase K (37 °C, 30 min) followed by PBS washes. Sections were incubated with TUNEL reaction mixture (TdT:Biotin-dUTP = 1:9) at 37 °C for 60 min in the dark, followed by PBS washes and treatment with reaction stop solution. Streptavidin-HRP was then applied at 37 °C for 30 min, followed by additional PBS washes. Chromogenic development was performed with DAB for 30 s to 5 min, terminated with distilled water, and sections were counterstained with hematoxylin, differentiated in 1% acid alcohol, rinsed, dehydrated through graded ethanol, cleared in xylene, and mounted with neutral resin. Stained sections were examined and photographed under a light microscope (Han et al., 2022).

### 2.6 Wound healing assay

The wound healing assay mimics cell migration as it occurs *in vivo* and serves as a straightforward, cost-effective approach for evaluating migratory behavior *in vitro*. In this method, a deliberate gap—referred to as a “scratch”—is created on a confluent cell monolayer. Cells located at the borders of this gap progressively move inward, leading to closure of the scratched area. Images of the cells were taken at 0 and 24 h after the scratch was made (Wang et al., 2023).

### 2.7 Scanning electron microscope

Liver samples were first fixed with glutaraldehyde followed by 1% osmium tetroxide. After fixation, the tissues were dehydrated and embedded in Epon resin to prepare ultrathin sections measuring approximately 200–400 Å. These sections were then stained sequentially with uranyl acetate and lead citrate before being examined under a digital electron microscope.

### 2.8 Transmission electron microscope experiments

Following collection, the samples were immersed in 2.5% glutaraldehyde and fixed overnight at 4 °C. Afterward, they were rinsed and post-fixed with osmium acid, then subjected to a graded acetone dehydration process. The tissues were embedded in epoxy resin to produce ultrathin sections, which were subsequently stained, examined, and imaged using a transmission electron microscope.

### 2.9 MRNA sequencing analysis

After total RNA was isolated from the samples, mRNA molecules were captured and purified using oligo (dT) magnetic beads, then randomly sheared with a fragmentation reagent. PCR products ranging from 200 to 300 bp were separated and collected from a 6% polyacrylamide Tris-borate-EDTA gel to complete library construction. The resulting libraries were sequenced on an Illumina HiSeq 2500 platform, and the reads were aligned to the corresponding mRNA reference database. Differentially expressed genes were identified through Chi-square testing, followed by GO and KEGG analyses for functional enrichment.

### 2.10 RT-qPCR

Quantitative primers for the differentially expressed genes were designed using Primer Premier 6.0, with β-actin selected as the internal control. The qPCR reaction mixture consisted of 1 μL cDNA, 0.4 μL of each primer (10 μmol/L), 0.4 μL Rox reference dye II, 10 μL SYBR Green real-time PCR Master Mix, and 7.8 μL ddH_2_O. The amplification protocol included an initial denaturation at 95 °C for 15 s, followed by 40 cycles of 5 s at 95 °C and 34 s at 60 °C. After the reaction, melting curve analysis was performed, and relative expression levels were calculated using the 2^−ΔΔCT^ method. For protein expression analysis, lysates from BMECs were subjected to SDS-PAGE, transferred to nitrocellulose membranes (Millipore, United States of America), and incubated with primary antibodies, including a rabbit monoclonal anti-TGFB2 (19999-1-AP, Sanying, Wuhan, China) or a mouse monoclonal anti-β-actin (66009-1-IG, Proteintech, China).

### 2.11 Statistical analysis

Statistical analyses were performed using SPSS 18.0, and the results were visualized with GraphPad Prism V5.0. A P-value <0.05 was considered statistically significant.

## 3 Results

### 3.1 Effects of CdCl_2_ exposure on serum biochemical parameters and peripheral leukocyte profiles in mice

**FIGURE 1:**
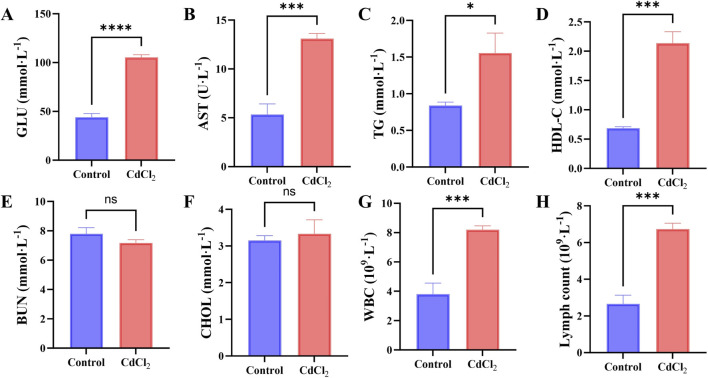
Effects of CdCl_2_ treatment on serum biochemical parameters and peripheral leukocyte counts in mice. **(A–D)** Serum levels of glucose (GLU), aspartate aminotransferase (AST), triglycerides (TG), and high-density lipoprotein cholesterol (HDL-C) in control and CdCl_2_-treated groups. **(E,F)** Serum levels of blood urea nitrogen (BUN) and total cholesterol (CHOL). **(G,H)** Counts of peripheral white blood cells (WBC) and lymphocytes. Data are presented as mean ± SD. *P < 0.05, **P < 0.01, ***P < 0.001, ****P < 0.0001 vs. control group.

Serum biochemical analysis revealed that CdCl_2_ exposure markedly disrupted hepatic function. Compared with the control group, mice treated with CdCl_2_ exhibited significant elevations in serum glucose (GLU), aspartate aminotransferase (AST), tri-glycerides (TG), and high-density lipoprotein cholesterol (HDL-C) levels (Figures 1A–D). Among these, AST—a key clinical indicator of hepatocellular injury—showed a pronounced increase, strongly suggesting the occurrence of acute hepatocellular damage. The abnormal increases in GLU, TG, and HDL-C further indicate that Cd exposure substantially impaired hepatic metabolic processes. In contrast, blood urea nitrogen (BUN) and total cholesterol (CHOL), which primarily reflect renal function and lipid metabolism, respectively, did not differ significantly between the control and treated groups (Figures 1E,F). These findings suggest that, under the present experimental conditions, the toxic effects of Cd were predominantly localized to the liver.

In addition, to evaluate whether Cd exposure elicited a systemic inflammatory response, peripheral white blood cell (WBC) and lymphocyte (Lymph) counts were examined. The results showed that CdCl_2_ treatment significantly increased both parameters (Figures 1G,H), indicating the activation of a typical inflammatory response in the organism.

### 3.2 Hematological alterations and histopathological changes in the liver induced by CdCl_2_ treatment

**FIGURE 2:**
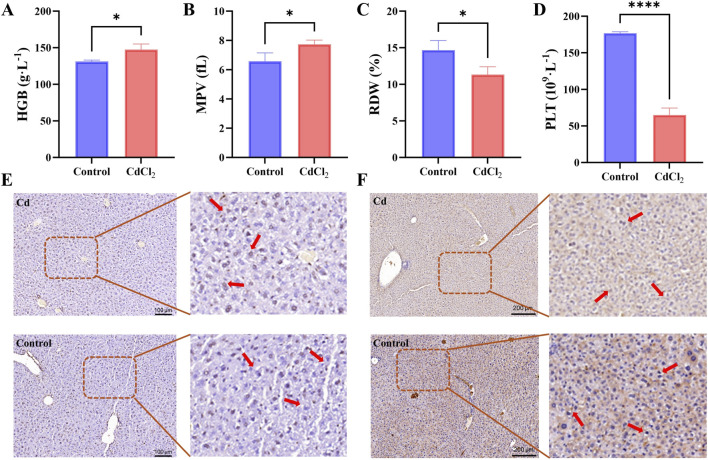
Hematological alterations and histological evidence of liver injury induced by CdCl_2_ treatment. **(A–D)** Hematological parameters: hemoglobin (HGB), mean platelet volume (MPV), red blood cell distribution width (RDW), and platelet count (PLT) in both groups. **(E)** TUNEL staining of liver tissue sections. Apoptotic hepatocytes are characterized by brown-stained nuclei (indicated by arrows), while normal hepatocytes display blue nuclei. The right panel shows a magnified view of the boxed area. **(F)** Immunohistochemical staining for PTX3 expression in liver tissues. Positive PTX3 expression is observed as brown or brownish-yellow staining in the cytoplasm of hepatocytes (indicated by arrows). The right panel shows a magnified view of the boxed area. Scale bar = 200 μm. Representative images from at least three independent mice per group are shown. *P < 0.05, ****P < 0.0001 vs. control group.

At the hematological level, mice exposed to CdCl_2_ exhibited a significant decrease in hemoglobin (HGB), platelet (PLT), and mean platelet volume (MPV) levels compared with the control group (Figures 2A,B,D). In addition, red blood cell distribution width (RDW) was also markedly reduced following CdCl_2_ exposure (Figure 2C). To further validate Cd-induced hepatic injury at the histological level, TUNEL staining was performed on liver tissues. Minimal apoptotic signals were observed in the control group, whereas extensive brown-positive staining was evident in the CdCl_2_-treated group, indicating that cadmium exposure markedly induced large-scale hepatocellular apoptosis (Figure 2E). To confirm the local inflammatory status of the liver, immunohistochemical (IHC) analysis was conducted to detect the expression of the inflammatory marker pentraxin-3 (PTX3). As shown in Figure 2F, PTX3 expression was markedly upregulated in the liver tissues of CdCl_2_-treated mice, confirming at the protein level that severe inflammatory responses occurred in hepatic tissue following cadmium exposure.

### 3.3 Cadmium directly disrupts hepatocellular structure and impairs the liver’s regenerative capacity

**FIGURE 3:**
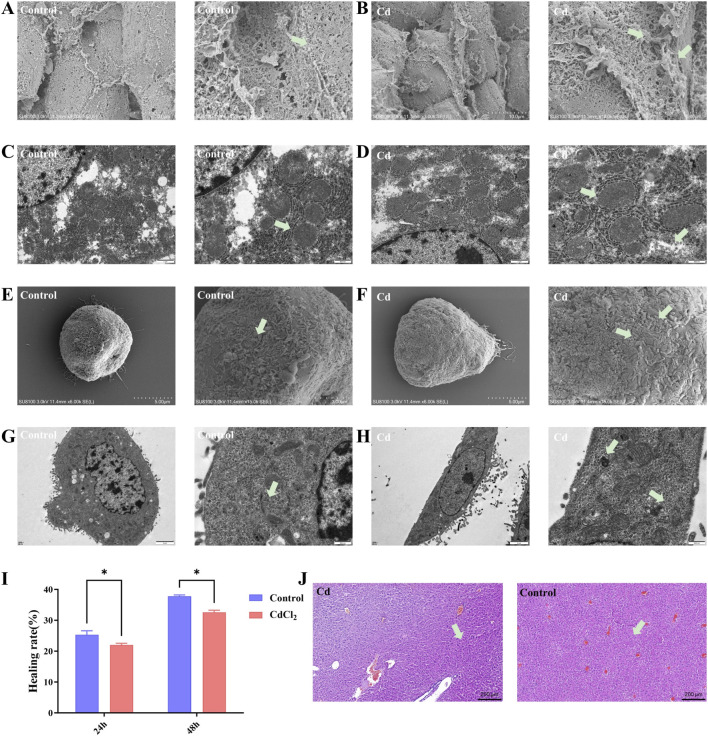
Effects of cadmium on the microstructure of hepatocytes and *in vitro* cell function. **(A,B)** SEM images of liver tissue samples. **(A)** Control group: Arrows indicate the surface of normal hepatocytes with intact cellular connections. **(B)** CdCl_2_ group: Arrows point to the disrupted hepatocyte surface and disordered cellular arrangement induced by cadmium. **(C,D)** TEM images of liver tissue samples. **(C)** Control group: Arrows indicate normal mitochondria with dense matrix and clear cristae structure. **(D)** CdCl_2_ group: Arrows highlight swollen mitochondria characterized by vacuolization and cristolysis (disappearance of cristae). **(E,F)** SEM images of *in vitro* cultured AML-12 hepatocytes. **(E)** Control group: Arrows indicate abundant and dense microvilli covering the cell surface. **(F)** CdCl_2_ group: Arrows indicate loss of microvilli and surface smoothness/blebbing, suggesting cytoskeletal damage. **(G,H)** TEM images of *in vitro* cultured AML-12 hepatocytes. **(G)** Control group: Arrows point to healthy mitochondria and endoplasmic reticulum. **(H)** CdCl_2_ group: Arrows indicate mitochondrial swelling and the formation of intracellular vacuoles. **(I)** Wound healing assay to detect the healing rate of AML-12 cells under different treatments. **(J)** H&E staining of liver tissues. Left Panel (Cd): Arrows indicate focal inflammatory cell infiltration and necrotic hepatocytes with pyknotic nuclei. Right Panel (Control): Arrows indicate normal hepatic cords and central vein structure without inflammatory changes. Scale bar = 200 μm.

Hematoxylin and eosin (H&E) staining provided a direct visualization of Cd-induced histopathological alterations in liver tissues. As shown in Figure 3J, liver sections from control mice displayed normal lobular architecture, well-organized hepatic cords, and morphologically intact hepatocytes. In contrast, CdCl_2_-treated mice exhibited pronounced pathological changes, including disordered hepatic cord arrangement, extensive hepatocellular necrosis, and marked inflammatory cell infiltration. Scanning electron microscopy (SEM) revealed that hepatocytes in the control group exhibited a smooth surface and well-defined morphology (Figure 3A), whereas those in the CdCl_2_-treated group displayed rough and uneven surfaces, loosened intercellular junctions, and severely distorted cellular morphology (Figure 3B). Transmission electron microscopy (TEM) analysis demonstrated that hepatocytes in the control group possessed well-defined and intact organelles (Figure 3C). In contrast, hepatocytes from the CdCl_2_-treated group showed swollen mitochondria, dilated and fragmented endo-plasmic reticulum, and blurred intracellular structures, indicating profound subcellular damage (Figure 3D).

We further performed in parallel in a Wound healing assay using the mouse hepatocyte cell AML-12 to validate the *in vivo* findings. Consistent with the results observed in liver tissues, SEM analysis revealed that CdCl_2_-treated AML-12 cells changed from a normal adherent and well-spread morphology to a contracted and rounded shape, with numerous folds and microvilli appearing on the cell surface (Figures 3E,F). TEM observations similarly demonstrated that CdCl_2_ exposure caused severe destruction of intracellular organelles in AML-12 cells (Figures 3G,H). To determine whether these structural alterations further impaired the cells’ migratory capacity—which is critical for hepatic self-repair—we performed a wound-healing assay to simulate the regenerative process following tissue injury. As shown in Figure 3I, control AML-12 cells exhibited robust migratory activity and almost completely closed the scratch area within 48 h, whereas CdCl_2_-treated cells displayed markedly reduced migration, resulting in a significantly lower wound closure rate compared with the control group.

### 3.4 Association between cadmium-induced liver injury and hepatic inflammatory response

**FIGURE 4:**
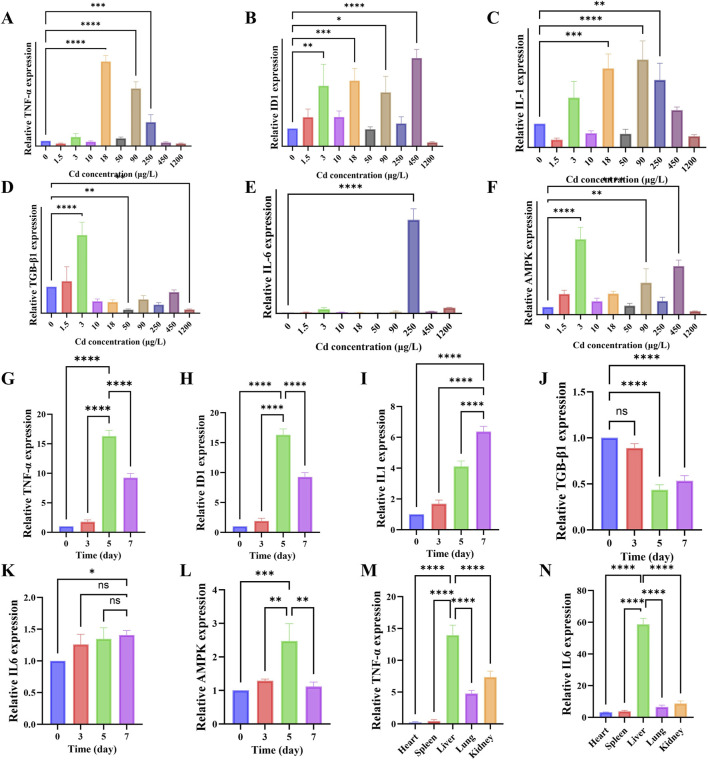
Analysis of cadmium-induced expression of inflammation-related genes in the liver. **(A–F)** Relative mRNA expression levels of inflammation-related genes (TNF-α, ID1, IL-1, TGF-β1, IL-6, AMPK) in the liver after treatment with different concentrations of CdCl_2_. **(G–L)** Relative mRNA expression levels of the above inflammation-related genes in the liver after treatment with a fixed concentration of CdCl_2_ for different durations. **(M,N)** Comparison of the relative mRNA expression levels of TNF-α and IL-6 in different organs (heart, liver, spleen, lung, kidney).

In this study, the effects of cadmium exposure on inflammation-associated molecules were systematically investigated from three perspectives—dose, exposure duration, and tissue specificity. In the dose-dependent study, mice were administered varying concentrations of CdCl_2_, and the mRNA levels of key inflammatory mediators were quantified by qPCR. The results revealed that multiple pro-inflammatory cytokines were markedly upregulated within the medium-to-high dose range, but their expression patterns exhibited nonlinear dynamics. Specifically, Both TNF-α and IL-1 showed pronounced expression peaks at particular exposure doses of CdCl_2_ (Figures 4A,C), whereas IL-6 was predominantly induced at higher doses (Figure 4E). In addition, ID1 and AMPK, both associated with inflammatory and stress responses, were elevated mainly at high concentrations, while TGF-β1 did not show a monotonic increase and instead fluctuated or declined at low-to-moderate doses (Figures 4B,D,F). These findings indicate that Cd-induced inflammation follows a threshold- or peak-type response pattern, rather than a simple dose-dependent enhancement. In the time-course experiment, mice were treated with CdCl_2_ (18 μg/L, 0.8 mL/day), and liver samples were collected at days 3, 5, and 7 post-exposure. Line-plot analyses of qPCR data demonstrated that the expression of key inflammatory cytokines—TNF-α, IL-1, and IL-6—progressively increased over time, reaching maximal levels at either day 5 or day 7 (Figures 4G–L). This temporal pattern suggests that Cd-induced hepatic inflammation represents a progressive and sustained pathological process, rather than a transient stress response. Finally, to explore tissue specificity, we compared inflammatory responses across major organs, including the heart, liver, spleen, lung, and kidney. Total RNA was extracted from each tissue, and the expression levels of TNF-α and IL-6 were quantified. Remarkably, both cytokines exhibited several-to dozens-fold higher mRNA expression in the liver compared with other organs (Figures 4M,N).

### 3.5 miRNA transcriptomics reveal the regulatory role of miRNAs in Cd-Induced hepatic injury

**FIGURE 5:**
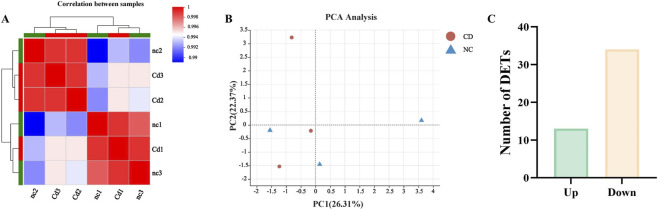
Global profiling of hepatic miRNA expression following Cd exposure. **(A)** Heatmap of inter-sample correlation. **(B)** Principal Component Analysis (PCA) plot. **(C)** Statistical plot of differentially expressed miRNAs.

The initiation and precise regulation of inflammatory responses rely on complex molecular networks, among which microRNAs (miRNAs) act as pivotal post-transcriptional regulators. By targeting multiple inflammation-related genes, miRNAs play central roles in either amplifying or suppressing inflammatory signaling. To elucidate the upstream regulatory mechanisms underlying Cd-induced hepatic inflammation, we performed high-throughput miRNA sequencing on liver tissues from control and CdCl_2_-treated mice. Correlation heatmap analysis revealed strong intragroup consistency and clear inter-group separation, indicating high reproducibility and distinct expression profiles between treatment conditions (Figure 5A). Furthermore, principal component analysis (PCA) demonstrated that samples from the control and CdCl_2_-treated groups clustered into two well-separated groups, confirming that cadmium exposure induced a global alteration in the hepatic miRNA expression landscape (Figure 5B).

**FIGURE 6:**
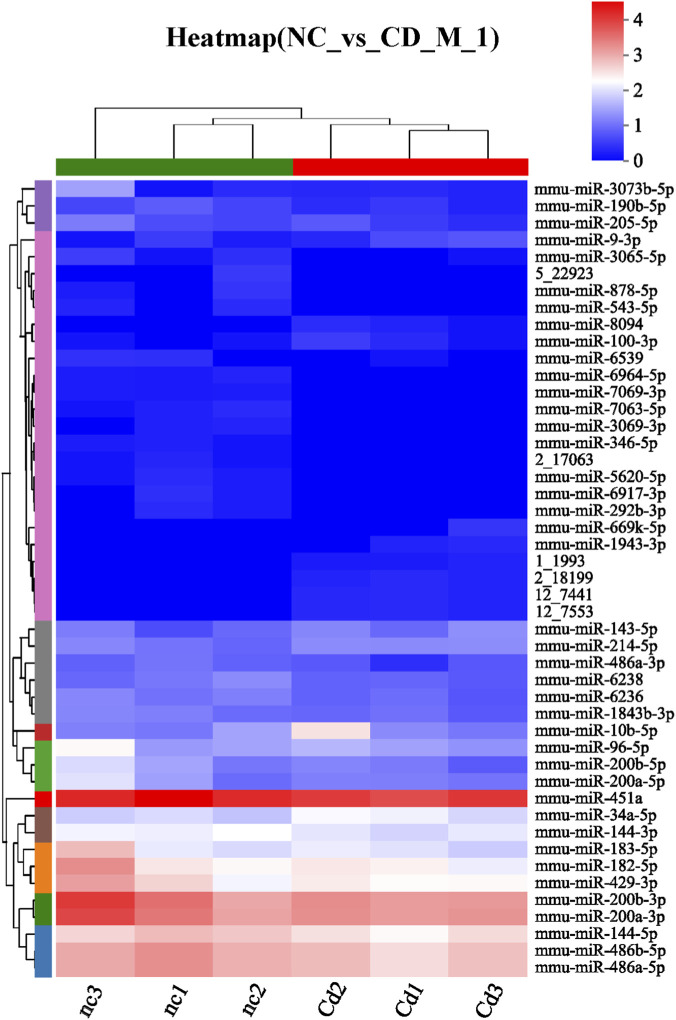
Clustering heatmap of differentially expressed miRNAs.

**FIGURE 7:**
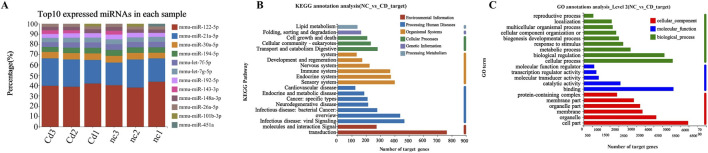
Functional annotation of differentially expressed miRNAs and their target genes. **(A)** Top 10 most abundant miRNAs in each sample. **(B)** KEGG functional annotation of target genes of differentially expressed miRNAs. **(C)** GO functional annotation of target genes of differentially expressed miRNAs.

Based on high-quality sequencing data, we identified a set of differentially ex-pressed miRNAs (DE-miRNAs) that exhibited significant changes following Cd expo-sure. A total of 47 DE-miRNAs were detected, of which 13 were significantly upregulated and 34 were downregulated (Figure 5C). The hierarchical clustering heatmap clearly illustrated distinct expression patterns between the control and CdCl_2_-treated groups (Figure 6). Moreover, the top 10 most abundantly expressed miRNAs in each group were ranked and visualized to highlight dominant miRNA species under different conditions (Figure 7A). To further explore the biological significance of these DE-miRNAs, their predicted target genes were subjected to Gene Ontology (GO) annotation and Kyoto Encyclopedia of Genes and Genomes (KEGG) pathway enrichment analyses. GO enrichment analysis indicated that the target genes were mainly associated with cellular processes, action potential, and sodium ion transmembrane transport (Figures 7C, 9B). In parallel, KEGG pathway analysis revealed a marked enrichment of these target genes in the MAPK signaling pathway (Figures 7B, 9A).

**FIGURE 8:**
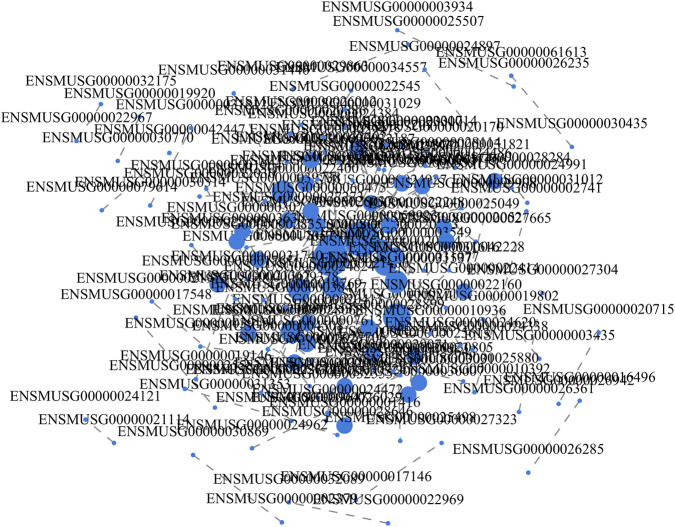
Protein-protein interaction (PPI) network of target genes. Nodes represent individual genes, and edges indicate known or predicted interactions. Key hub genes with high network centrality are highlighted, indicating their potential importance in regulating cadmium-induced hepatotoxicity.

**FIGURE 9:**
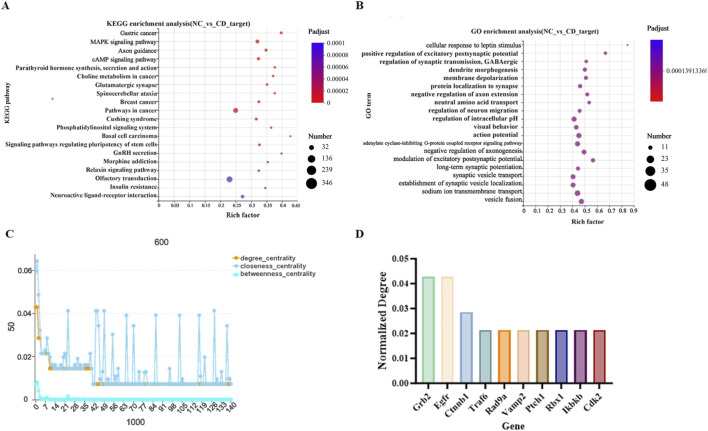
Enrichment analysis and protein–protein interaction (PPI) network of miRNA target genes. **(A)** KEGG enrichment analysis of target genes of differentially expressed miRNAs. **(B)** GO enrichment analysis of target genes of differentially expressed miRNAs. **(C,D)** PPI network centrality analysis and screening of core hub genes.

To identify the most critical regulatory nodes within the complex target gene network, we constructed a protein–protein interaction (PPI) network based on the predicted target genes and performed centrality analysis. The PPI network visualization (Figure 8) revealed intricate interaction relationships among the target proteins. By calculating the centrality coefficients of each node (Figure 9C), we identified the top 10 hub genes, including Grb2, Egfr, Ctnnb1, Traf6, Rad9a, Vamp2, Ptch1, Rbx1, Ikbkb, and Cdk2 (Figure 9D). These genes participate in diverse but essential biological processes: Grb2 functions as an adaptor protein integrating multiple signaling pathways; Egfr regulates cell proliferation, differentiation, and survival; Ctnnb1 encodes β-catenin, a core component of the classical cadherin adhesion complex; Traf6 mediates inflammatory and immune signaling through K63-linked polyubiquitin chain formation; Rad9a regulates cell-cycle checkpoints; Vamp2 controls vesicle fusion and neuro-transmitter release; Ptch1 governs cell growth, differentiation, and tissue development; Rbx1 participates in protein ubiquitination and SCF complex assembly; Ikbkb is a key modulator of the NF-κB pathway and inflammatory responses; and Cdk2 plays a central role in cell-cycle progression. These genes occupy key “traffic hub” positions within the regulatory network, and their dysregulation may exert decisive effects on overall network stability and biological function.

## 4 Discussion

Cadmium (Cd) is among the most toxic heavy metals and poses substantial environmental and health risks due to its bioaccumulation and strong reactivity (Ju et al., 2017). The liver, as a primary organ for Cd metabolism, is vulnerable to Cd-induced necroinflammation and fibrosis (Liu et al., 2024; Hyder et al., 2013; Go et al., 2015). Previous studies have largely focused on cadmium-induced structural damage to the liver and the accompanying inflammatory responses. In this study, instead of reiterating previously de-scribed pathological features, we emphasize that Cd exposure initiates a coordinated hepatocellular stress response. The concurrent appearance of inflammatory activation, organelle dysfunction, and apoptosis suggests that these processes are mechanistically interconnected rather than independent outcomes. Such integration suggests that Cd-induced acute liver injury may involve a reinforcing interplay among multiple cellular stress pathways, providing additional mechanistic insights beyond the characterization of established biochemical and histological alterations. More importantly, our findings demonstrate that cadmium directly targets hepatocytes, and the observed liver injury is not merely a consequence of systemic inflammatory or indirect effects (Figures 1–3). Furthermore, the wound-healing assay revealed that Cd exposure markedly inhibited hepatocyte migratory capacity, suggesting a suppression of the liver’s intrinsic regenerative potential, which may contribute to the persistence and progression of tissue damage (Figure 3). Although previous studies have noted the inflammatory potential of Cd exposure (Wen et al., 2025; Liu J. et al., 2025; Liu H. et al., 2025; Li Y. et al., 2025), such observations have largely been fragmented or correlative. In contrast, our study clearly demonstrates that Cd-induced hepatic inflammation exhibits strict dose- and time-dependence (Figure 4). Although the dose-response analysis revealed the unexpected finding that 18 μg/mL Cd induced the most severe hepatic injury, our overall results consistently demonstrate that inflammatory mediators such as TNF-α and IL-6 not only serve as reliable biomarkers indicating the severity of Cd-induced liver damage, but may also represent potential therapeutic targets for mitigating heavy metal–induced hepatotoxicity.

**FIGURE 10:**
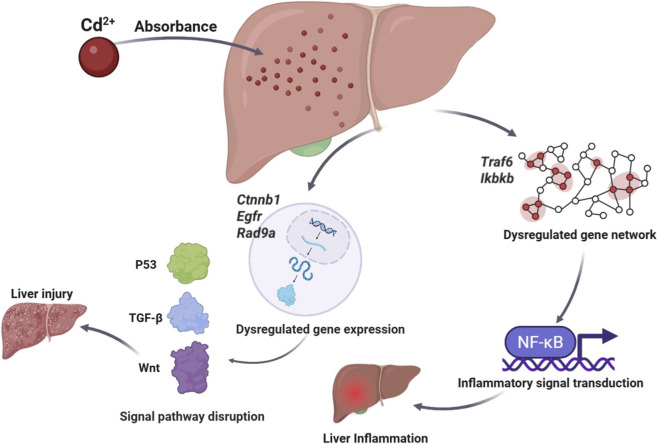
Mechanism diagram of Cd-induced liver damage.

To elucidate the molecular “switch” contributing to hepatic inflammation, we focused on microRNAs (miRNAs), important regulators of post-transcriptional gene expression. Our miRNA-omics analysis identified a set of differentially expressed miRNAs and revealed potential associations between these alterations and inflammatory or apoptotic phenotypes. However, these data represent correlative patterns rather than demonstrated mechanistic drivers. Although GO and KEGG analyses indicated enrichment in pathways such as MAPK signaling, these findings should be interpreted as hypothesis-generating rather than confirming that miRNAs causally initiate Cd-induced inflammation. Following Cd exposure, a robust immune-inflammatory response was triggered in the mouse liver. The expression of proinflammatory cytokines including TNF-α and IL-6 increased significantly in a dose- and time-de (Li K. et al., 2025) pendent manner, indicating that Cd activates inflammatory signaling through the TNF/NF-κB and MAPK pathways (Kim et al., 2024; Ni et al., 2024; Song et al., 2025). Similar observations have been reported by Liu et al., who demonstrated that Cd exposure activates the MAPK cascade during atherosclerotic plaque formation (Liu H. et al., 2025), supporting the pivotal role of this path-way in Cd-induced inflammation. Concurrently, our miRNA sequencing data revealed extensive miRNA dysregulation, suggesting that Cd disrupts the hepatic miRNA regulatory network and thereby alters multiple key molecular cascades. On one hand, several inflammation-related hub genes—such as Traf6 and Ikbkb—were upregulated following Cd exposure. Although their predicted upstream miRNAs were concurrently downregulated, this regulatory relationship remains inferential. The observed gene expression patterns are compatible with strengthened NF-κB signaling, but direct evidence of miRNA–mRNA interaction requires functional validation. On the other hand, genes involved in hepatocyte survival and apoptosis regulation—including Ctnnb1 (β-catenin), Egfr, and Rad9a—also showed altered expression. These changes suggest possible involvement of Wnt, TGF-β, and p53 pathways, although further experiments are needed to determine whether these alterations are causal contributors or secondary responses to Cd-induced stress. As a result, the liver becomes simultaneously subjected to uncontrolled immune-mediated inflammation and accelerated apoptotic progression. This ultimately results in extensive hepatocyte death, necrosis, and impaired hepatic regenerative capacity (Figure 10). Collectively, these pathological cascades culminate in acute hepatic failure, characterized by elevated serum transaminase levels and severe histological damage (Figure 3).

In summary, this study integrates miRNA-omics and molecular validation experiments to construct a post-transcriptional regulatory landscape of Cd-induced hepatic injury. Our findings suggest that Cd toxicity can be conceptualized as an miRNA-mediated inflammatory–apoptotic positive feedback loop, driven by the synergistic effects of liver-specific inflammatory activation and disruption of miRNA homeo-stasis. This finding deepens our understanding of the molecular mechanisms under-lying heavy metal–induced hepatotoxicity and provides new potential targets and strategies for the intervention of Cd-induced liver injury. miRNAs have emerged as a promising class of therapeutic targets for liver diseases, and numerous studies have begun to explore their clinical potential in this field, for example, by transferring miR-1275 to alleviate hepatic ischemia–reperfusion injury, applying miR-190b-5p and miR-296-3p for the treatment of liver fibrosis, and modulating miR-155-5p to improve alcohol-induced liver injury (Fan et al., 2025; Gong et al., 2025; Markovic et al., 2025; Ma et al., 2025; Li W. et al., 2025; Li S. et al., 2025; Zheng et al., 2025). Based on the newly identified targets in our study, it may be possible to develop miRNA-based therapeutics that specifically modulate key regulatory miRNAs to alleviate Cd-induced hepatic inflammation. This study has several limitations. First, the acute Cd exposure model may not fully recapitulate chronic low-dose environmental exposure scenarios. Second, whole-liver miRNA profiling prevents distinguishing hepatocyte-specific alterations from changes occurring in non-parenchymal cells. Third, without gain- or loss-of-function experiments, the regulatory roles of specific miRNAs remain inferential. Future work should include functional validation of candidate miRNAs using hepatocyte-specific overexpression or silencing models, as well as temporal and single-cell approaches to determine whether miRNA dysregulation acts upstream or downstream of inflammatory activation during Cd-induced liver injury.

## 5 Conclusion

This study provides evidence that a liver-centered inflammatory cascade, potentially modulated by a specific miRNA regulatory network, contributes to the pathophysiology of cadmium (Cd)-induced acute liver injury. Several of the identified miRNAs may represent candidate targets for further investigation in the context of liver disease; however, their functional roles and therapeutic potential require validation in future mechanistic studies.

## Data Availability

The datasets presented in this study can be found in online repositories. The names of the repository/repositories and accession number(s) can be found in the article/supplementary material.
